# Systemic Bile Acids Affect the Severity of Acute Pancreatitis in Mice Depending on Their Hydrophobicity and the Disease Pathogenesis

**DOI:** 10.3390/ijms232113592

**Published:** 2022-11-05

**Authors:** Quang Trung Tran, Matthias Sendler, Mats L. Wiese, Julia Doller, Lukas Zierke, Marcel Gischke, Juliane Glaubitz, Van Huy Tran, Michael Lalk, Uwe T. Bornscheuer, Frank Ulrich Weiss, Markus M. Lerch, Ali A. Aghdassi

**Affiliations:** 1 Department of Internal Medicine A, University Medicine Greifswald, Ferdinand-Sauerbruch-Straße, 17475 Greifswald, Germany; 2 Department of Internal Medicine, University of Medicine and Pharmacy, Hue University, Hue City 530000, Vietnam; 3 Institute of Biochemistry, University Greifswald, 17489 Greifswald, Germany; 4 Ludwig Maximilian University Hospital, Ludwig Maximilian University of Munich, 81377 Munich, Germany

**Keywords:** acute pancreatitis, bile acids, CCK1R binding, hydrophobicity

## Abstract

Acute pancreatitis (AP) is a major, globally increasing gastrointestinal disease and a biliary origin is the most common cause. However, the effects of bile acids (BAs), given systemically, on the pancreas and on disease severity remains elusive. In this study, we have investigated the roles of different circulating BAs in animal models for AP to elucidate their impact on disease severity and the underlying pathomechanisms. BAs were incubated on isolated acini and AP was induced through repetitive injections of caerulein or L-arginine; pancreatic duct ligation (PDL); or combined biliopancreatic duct ligation (BPDL). Disease severity was assessed using biochemical and histological parameters. Serum cholecystokinin (CCK) concentrations were determined via enzyme immunoassay. The binding of the CCK1 receptor was measured using fluorescence-labeled CCK. In isolated acini, hydrophobic BAs mitigated the damaging effects of CCK. The same BAs further enhanced pancreatitis in L-arginine- and PDL-based pancreatitis, whereas they ameliorated pancreatic damage in the caerulein and BPDL models. Mechanistically, the binding affinity of the CCK1 receptor was significantly reduced by hydrophobic BAs. The hydrophobicity of BAs and the involvement of CCK seem to be relevant in the course of AP. Systemic BAs may affect the severity of AP by interfering with the CCK1 receptor.

## 1. Introduction

Acute pancreatitis (AP) is one of the most frequent non-malignant gastroenterological disorders requiring hospitalization. In recent decades, its incidence has increased steadily worldwide, with an annual aggregate cost of more than USD 2.63 billion in the United States [1,2]. Approximately one fifth of AP patients suffer from local or systemic complications and (multi-)organ failure, with a mortality rate of up to 40% in the most severe cohort [3]. Despite intensive efforts, no causal treatment is available for this condition, and therefore, management is solely based on symptomatic and supportive therapy or dealing with its complications [4]. AP is considered to originally occur in the pancreatic acinar cells, which are vulnerable to extracellular pathological stimuli [5]. In these exocrine units, digestive proteases, beginning with trypsin [6], are prematurely activated after co-localization with the lysosomal hydrolase cathepsin B. Imbalances between the activation and degradation of digestive enzymes may result in injury of the acini [7]. Among various etiologies, migrating gallstones are one of the most common causes of AP, which accounts for 30–50% of all cases [8].

Bile acids (BAs), the largest component of bile juice, have recently gained attention for their pathological role in both rodents and humans [9]. Starting from cholesterol, primary BAs are synthesized and subsequently conjugated in the liver with glycine or taurine [10]. Two major primary BAs are cholic acid and chenodeoxycholic acid. After secretion into the duodenum, they are metabolized to the secondary BAs, lithocholic acid and deoxycholic acid, by the gut microbiome. BAs in the intestine are reabsorbed, mainly in the distal ileum, and finally, return to the liver via enterohepatic circulation for recycling. In rodents, taurine-conjugated forms such as taurocholic acid and taurodeoxycholic acid are predominant [11,12,13]. BAs can also be classified according to their chemical properties. Depending on the hydroxylation, BAs are differentiated by their hydrophobicity index with different biological effects. There are, in total, approximately 20 different BAs, including primary compounds and their taurine- as well as glycine-conjugated forms, with hydrophobicity indices from −0.84 to +1.23. The more hydrophobic BAs are present, the higher the hydrophobicity indices, and vice versa. Among them, lithocholic acid (LCA) is one of the most hydrophobic agents [14,15]. Noticeably, taurolithocholic acid 3-sulfate (TLCS) is a strongly hydrophobic compound and was previously used in other studies on AP [16,17,18]. Tauroursodeoxycholic acid (TUDCA) is a typical hydrophilic BA and was approved for the treatment of some biliary disorders such as primary sclerosis cholangitis. Moreover, an emerging role of TUDCA is discussed for many other diseases, including AP [19,20,21], which makes this BA an attractive candidate for further investigations.

In addition to their ability to emulsify lipids, BAs show more and more clear effects in regulating many pathological processes [22]. The pathogenetic effects of some BAs have been reported for several diseases such as liver, biliary and metabolic disorders [9,23]. However, in pancreatitis, knowledge of the role of BAs is limited and mainly based on retrograde ductal infusion models, which remain controversial as the extent of the intra-pancreatic duct pressure and characteristics of infused agents also seem to contribute to acute inflammation [24]. Although it has been shown that AP is highly prevalent among hepatobiliary disorders [25], data on susceptibility to the development and modulation of pancreatitis in cholestatic disorders that are accompanied by increased serum levels of BAs are insufficient. In particular, the mechanisms through which BAs affect acinar cells, when given systemically, have not been elucidated.

This study aims to investigate the impact of various BAs using in vitro-to-in vivo models and addresses the underlying mechanisms through which BAs modulate the severity of AP. 

## 2. Results

### 2.1. Impact of Hydrophobic and Hydrophilic BAs in Mouse Isolated Acini

**Figure 1:**
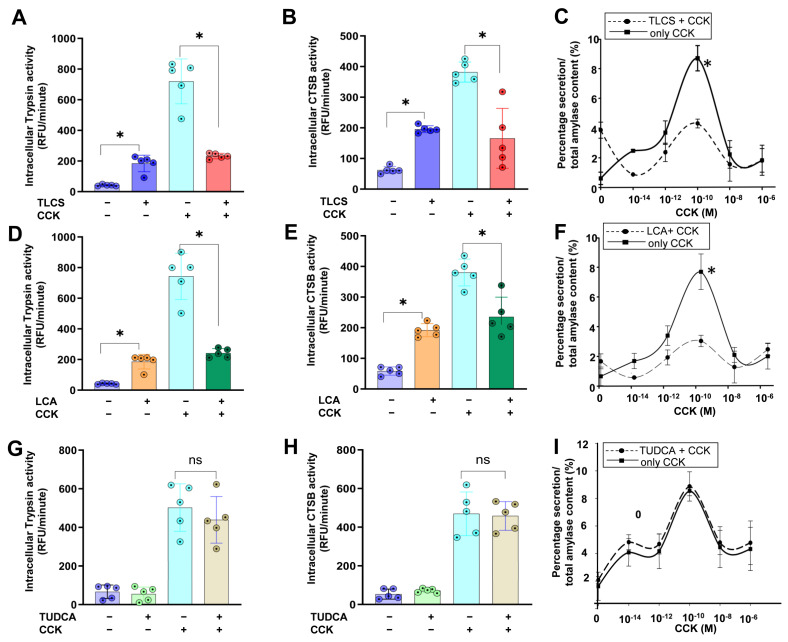
**Impact of BAs in CCK-stimulated acini.** (**A**,**B**) TLCS alone, a hydrophobic BA, induced intracellular protease activation, as shown for trypsin and cathepsin B, which was less prominent than with supramaximal CCK (1 µM). Co-incubation of CCK and TLCS attenuated the protease activation. (**C**) Stimulation of isolated acini with different concentrations of CCK showed a peak at 100 pM, which was blocked after co-incubation with TLCS. (**D**–**F**) Similarly, LCA alone, another hydrophobic BA showed the same results as TLCS in stimulating acinar cells and mitigating the impact of CCK. (**G**–**I**) In contrast, the hydrophilic TUDCA neither altered intracellular protease activation, nor decreased amylase secretion following co-incubation of CCK and TUDCA. Concentration for TLCS, LCA and TUDCA was 500 µM. All results were based on 5 experiments per group. Statistically significant differences for more than 3 groups were tested via one-way ANOVA followed by Tukey’s multiple comparison test, and significance levels of *p* < 0.05 are marked by an asterisk. ns: non significant; RFU: Relative Fluorescence Units.

We first investigated protease activation on the cellular level in isolated acini upon exposure to BAs with different levels of hydrophobicity. We initially performed in vitro experiments with lower concentrations (50, 100 and 200 µM) of BAs but no clear changes in intracellular protease activation were observed. The intracellular activities of trypsin and cathepsin B (CTSB), a known trypsinogen activator, were significantly higher when treated with 500 µM TLCS or LCA in comparison to the control cells. Interestingly, these hydrophobic BAs did not further enhance the effect of CCK in isolated living acini, and co-incubation of them with supramaximal CCK (1 µM) reduced intracellular protease activation compared to CCK alone (Figure 1A,B,D,E). The stimulation of acini with submaximal CCK concentrations led to peak amylase secretion at a concentration of 100 pM. The addition of the hydrophobic bile acids TLCS and LCA attenuated the amylase secretion, showing a similar effect as under conditions of supramaximal CCK (Figure 1C,F). Conversely, the hydrophilic bile acid TUDCA did not alter intracellular protease activity in acini either in unstimulated conditions or after supramaximal doses of CCK (Figure 1G,H). In parallel, exposure to physiologic CCK concentrations did not modify amylase secretion (Figure 1I). 

**Figure 2:**
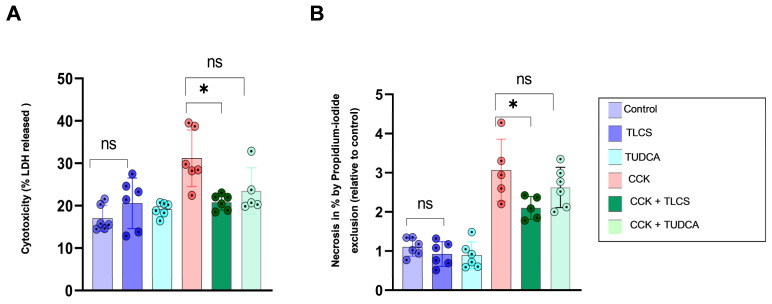
**Release of LDH and propidium iodide exclusion in mouse isolated acini.** Addition of bile acids TLCS and TUDCA in a final concentration of 500 µM to isolated acini did not increase LDH release (**A**) or propidium iodide exclusion (**B**), indicating no relevant cellular toxicity. Supramaximal CCK stimulation of acini increased both LDH release (**A**) and PI exclusion (**B**), which was reduced by TLCS but not TUDCA, compatible with protease activation in isolated acini. Graphs represent at least 5 animals per group. Statistically significant differences for more than 3 groups were tested via one-way ANOVA followed by Tukey’s multiple comparison test, and significance levels of *p* < 0.05 are marked by an asterisk. ns: non significant.

We further tested for the potentially toxic effects of BAs given at final concentrations of 500 µM on pancreatic acini; however, neither in the lactate dehydrogenase (LDH) (Figure 2A) nor the propidium iodide (PI) exclusion (Figure 2B) measurements did we observe an increase in cellular damage by BAs, which remained comparable to the control cells. Then, the addition of CCK enhanced both LDH release (Figure 2A) and PI exclusion (Figure 2B), indicating cell injury, which was reversed by hydrophobic but not hydrophilic BAs. 

These results suggest that hydrophobic but not hydrophilic BAs induced intracellular protease activation and mitigated the impact of CCK in isolated acini.

### 2.2. BAs Were Elevated in the Serum and Reached the Pancreas after Intravenous or Intraperitoneal Administration in Mice

**Figure 3:**
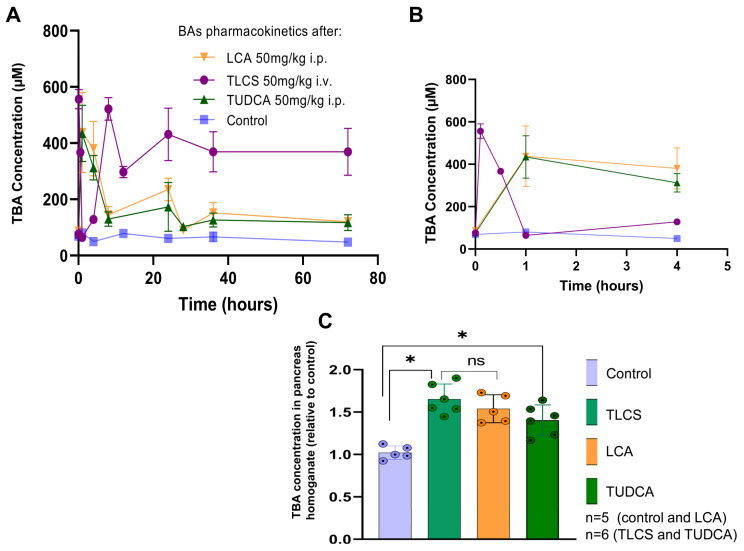
**Elevation of BAs in circulation and in pancreas of mice after systemic administration**. Pharmacokinetics of TLCS, LCA and TUDCA were assessed via measurement of total BA concentrations at different intervals after i.v. or i.p. injection up to 72 h. (**A**) After i.v. injection of TLCS, serum concentrations (purple line) increased quickly within 5 min, then decreased, and were cleared up from circulation after 60 min. A second peak occurred at 8 h, resulting from enterohepatic circulation through reabsorption, and serum concentrations slowly reduced during the monitoring time. Intraperitoneal injection of TUDCA (green line) or LCA (orange line) led to a delayed increase in serum concentrations, showing a maximum at around one hour. Concentrations then gradually decreased and were maintained at a slightly higher level than in controls (blue line). The mean absolute BA concentrations after administrating TLCS, LCA and TUDCA were 374.53 ± 21.07, 173.24 ± 12.25 and 164 ± 10.93 µM, respectively. (**B**) Illustration of serum BA concentrations during the first 4 h for clearer presentability. (**C**) All the injected BAs reached the pancreas, as shown by significant elevation of TBA concentration in pancreas homogenates. The mean value of TBA concentrations in the pancreas homogenate were 30.65 ± 2.73, 49.58 ± 5.33, 46.17 ± 4.95 and 42.13 ± 5.39 μM/mg protein for control mice, and TLCS-, LCA- and TUDCA-injected mice, respectively. Graphs represent at least 5 animals per group. Statistically significant differences for more than 3 groups were tested via one-way ANOVA followed by Tukey’s multiple comparison test, and significance levels of *p* < 0.05 are marked by an asterisk. ns: non significant.

Since the times for harvesting samples after inducing AP were 4 h in the caerulein model, 24 h in the duct ligation models and 72 h in the L-arginine model, we measured the total bile acid (TBA) concentrations in the serum and in the pancreas homogenates after intravenous (i.v.) or intraperitoneal (i.p.) injection up to three days. After tail vein injection of TLCS, serum concentrations increased quickly within 5 min, then, decreased and almost disappeared from circulation after one hour. Reabsorption through the enterohepatic circulation led to the second peak, which occurred at 8 h, and serum concentrations slowly reduced during the monitoring time. LCA and TUDCA were intraperitoneally injected, which led to slower increases in serum concentrations, showing a maximum at around one hour. The concentrations were then maintained at a higher level than in the controls (Figure 3A,B). The injected BAs, either i.v. or i.p., all reached the pancreas, as shown by the significant elevation of TBA concentration in pancreas homogenates for all three BAs (Figure 3C). The data demonstrate that BAs can reach the pancreas in an in vivo experiment either via i.v. or i.p. injection.

### 2.3. Hydrophobicity-Dependent Effects of BAs in CCK-Dependent Mouse AP Models

**Figure 4:**
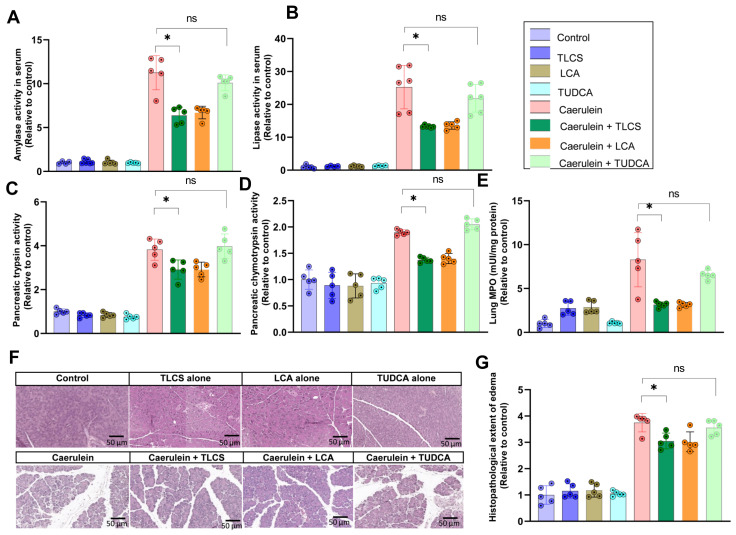
**Attenuated severity of caerulein-induced AP following hydrophobic BAs in mice.** (**A**,**B**) AP was induced by repetitive injections of caerulein, a cholecystokinin analogue. One hour or 30 min prior to the first caerulein injection, mice received a dose of 50 mg/kg TUDCA, LCA or TLCS. Serum amylase and lipase were elevated after 4 h of caerulein pancreatitis. Both enzyme activities decreased after pretreatment with TLCS or LCA, but not TUDCA. (**C**,**D**) We further determined pancreatic trypsin activity at 4 h and chymotrypsin activity at 1 h in pancreatic homogenates, which were both elevated in pancreatitis. Pretreatment with TLCS and LCA reduced these activities but TUDCA did not affect them in comparison with caerulein alone. (**E**) Lung MPO, an indicator of extra-pancreatic damage in AP, showed the same manner as pancreatic parameters, in which it was attenuated by TLCS as well as LCA, but not TUDCA, in caerulein-induced AP. (**F**,**G**) The corresponding histopathological damage was compatible with the biochemical indicators of AP, showing amelioration in the presence of hydrophobic TLCS or LCA and remaining unchanged in treatment with hydrophilic TUDCA. All graphs represent 4–6 animals per group. Statistically significant differences for more than 3 groups were tested via one-way ANOVA followed by Tukey’s multiple comparison test, and significance levels of *p* < 0.05 are marked by an asterisk. ns: non significant.

We next investigated the role of hydrophilic and hydrophobic BAs in an in vivo model dependent on the CCK analogue caerulein. Prior to caerulein administration, mice received an injection of TLCS, LCA or TUDCA (50 mg/kg bodyweight). Pancreatic damage was assessed through serological markers, protease activity in the pancreas homogenates, histology, and lung MPO for the determination of extra-pancreatic damage. While neither TUDCA, TLCS nor LCA alone had any effect in unstimulated mice, pretreatment with hydrophobic TLCS or LCA attenuated pancreatic injury, as observed for serum amylase (Figure 4A) and lipase (Figure 4B), as well as trypsin and chymotrypsin activities in the pancreatic homogenates (Figure 4C,D). Lung MPO measurements showed a decrease resulting from TLCS or LCA in caerulein-induced pancreatitis, indicating collateral attenuation of extra-pancreatic damage (Figure 4E), and local pancreatic damage was decreased after the addition of these hydrophobic BAs, as well (Figure 4F,G). In contrast, the hydrophilic TUDCA did not alter the activation of any of these parameters.

### 2.4. Aggravating Effects of Hydrophobic BAs in CCK-Independent AP Models in Mice

**Figure 5:**
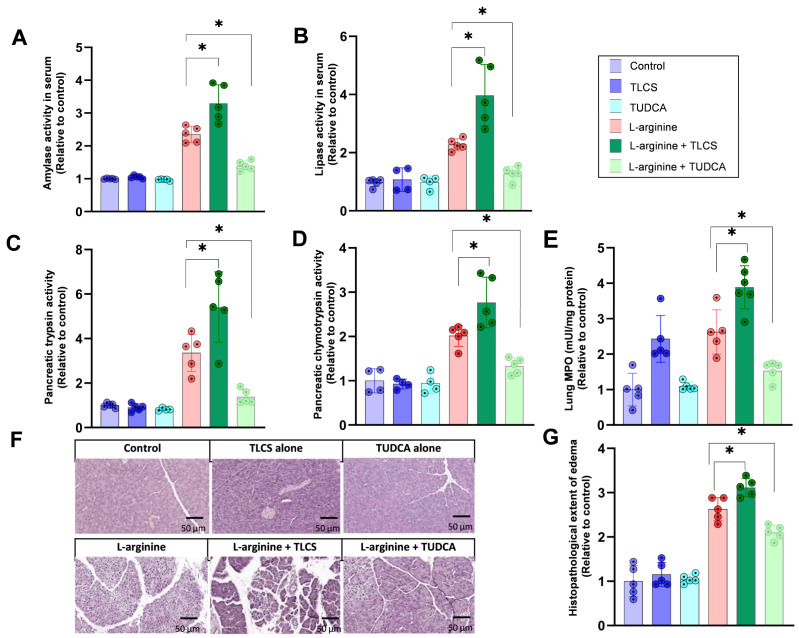
**Effects of BAs in L-arginine-induced AP in mice.** BAs were given via the same application route as for caerulein pancreatitis and mice were humanely killed after 72 h. (**A**,**B**) L-arginine induced AP, as shown by elevations of serum amylase and lipase after L-arginine injection. Pretreatment with TLCS caused disease aggravation, as shown by an increase in the enzyme activities, while TUDCA ameliorated disease severity. Neither TLCS nor TUDCA alone altered enzyme activities in the absence of L-arginine. (**C**,**D**) Pancreatic trypsin activity and chymotrypsin activity were enhanced in L-arginine pancreatitis. Protease activity was enhanced by TLCS pre-treatment and reduced when mice received TUDCA. (**E**) Lung MPO changed similarly to pancreatic damage, in which TLCS increased but TUDCA mitigated enzyme activity. (**F**,**G**) Organ damage, shown by histology in L-arginine pancreatitis, was more severe with TLCS but milder when TUDCA was added in comparison to L-arginine alone. All graphs represent 4–6 animals per group. Statistically significant differences for more than 3 groups were tested via one-way ANOVA followed by Tukey’s multiple comparison test, and significance levels of *p* < 0.05 are marked by an asterisk.

As our results from CCK- or CCK analogue-dependent experimental pancreatitis models indicate a reduction in severity in the presence of hydrophobic BAs, we were interested in the impact of BAs in conditions unrelated to CCK. L-arginine-induced pancreatitis is characterized by CCK-independent signaling pathways, which makes this model interesting for investigations of BA-related effects. In L-arginine-induced AP, there was maximal damage at 72 h, which was further enhanced by TLCS but ameliorated by TUDCA, as demonstrated by changes in serum amylase (Figure 5A) and lipase (Figure 5B) activities. Correspondingly, trypsin (Figure 5C) and chymotrypsin (Figure 5D) activities in pancreatic homogenates were elevated in L-arginine pancreatitis, indicating the activation of digestive proteases, and were even more enhanced after pretreatment with TLCS. On the other hand, TUDCA had an opposite effect (Figure 5C,D). Lung MPO enzyme activity for extra-pancreatic injury (Figure 5E) and local pancreatic damage, assessed via hematoxylin- and eosin-stained pancreatic sections, demonstrated aggravation of the disease under TLCS, while for TUDCA, a reversed effect was observed (Figure 5F,G).

**Figure 6:**
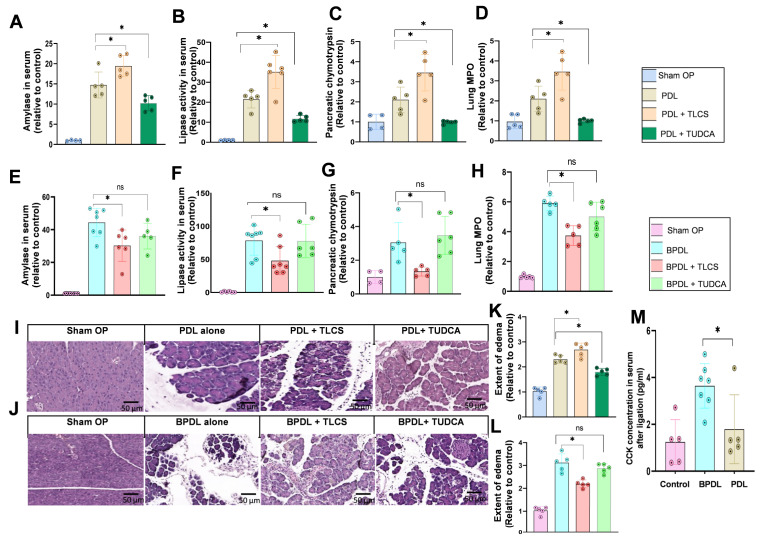
**Impact of BAs in mouse AP models based on duct ligation.** (**A**,**B**) Pancreatic duct ligation (PDL) caused a strong increase in serum amylase and lipase, which was further enhanced by pre-treatment with TLCS but mitigated by TUDCA. (**C**,**D**) Pancreatic chymotrypsin and lung MPO levels were higher when injecting additional TLCS but lower after treatment with TUDCA. (**E**–**H**) Ligation of both distal common bile duct and main pancreatic duct (BPDL) led to an increase in amylase, lipase, chymotrypsin and lung MPO, similar to the PDL model. However, pre-treatment with TLCS reduced enzyme activities, while the addition of TUDCA did not alter disease severity. (**I**,**K**) Organ injury, including the extent of edema in HE-stained slides, demonstrated increased severity with TLCS but attenuation with TUDCA in the PDL model for acute pancreatitis. (**J**,**L**) In contrast, local histological damage was reduced by TLCS when both the bile and the pancreatic duct were ligated. Additional TUDCA treatment did not show any effect compared to duct ligation alone. (**M**) Endogenous CCK levels were significantly higher in the BPDL compared to the PDL model, indicating involvement of CCK in the former. All graphs represent 4–7 animals per group. Statistically significant differences for more than 3 groups were tested via one-way ANOVA followed by Tukey’s multiple comparison test, and significance levels of *p* < 0.05 are marked by an asterisk. ns: non significant.

Disease severity and its dependence on BAs showed a similar pattern in the PDL model, which was independent of CCK as well. One day after ligation, AP was observed as shown by elevated levels of serum lipase, amylase, chymotrypsin in homogenates and lung MPO. Pretreatment with TLCS increased enzyme activities by about 25–40%, while there was attenuated severity under TUDCA (Figure 6A–D). In addition, the severity of histological damage correlated with findings from the enzyme measurements (Figure 6I,K).

Surprisingly, decreased severity of pancreatitis was observed in the BPDL model following TLCS injection. Both enzyme activities (Figure 6E–H) and organ damage were reduced (Figure 6J,L). This reduction resembled findings seen in the caerulein-induced pancreatitis model (Figure 4). On the other hand, TUDCA treatment did not alter disease severity in the caerulein model. Apparently, combined ligation of both ducts is related to CCK, as measurements of CCK concentrations in serum showed an almost three-fold increase after simultaneous ligation of the pancreatic and bile ducts after 30 min, while after pancreatic duct ligation alone, serum CCK remained unaffected (Figure 6M).

### 2.5. Interaction of Hydrophobic and Hydrophilic BAs with CCK1R on Mouse Isolated Acini

**Figure 7:**
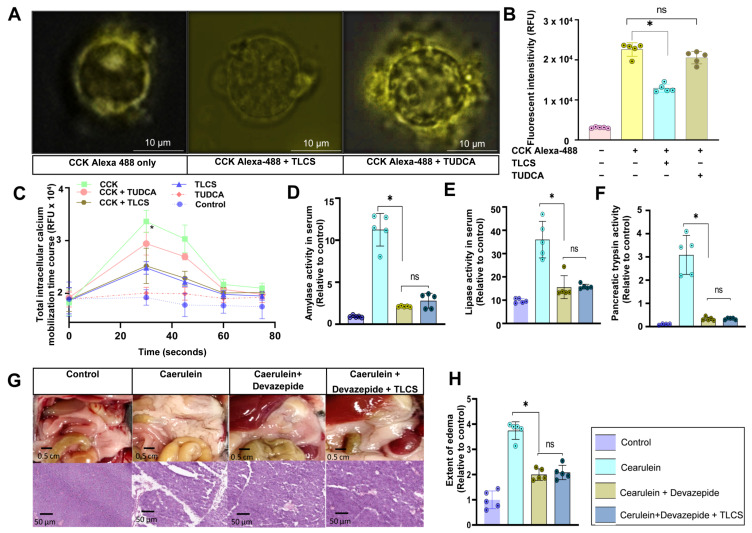
**Interaction of BAs with the CCK1 receptor and intracellular calcium release in mice.** (**A**) Fluorescence-labeled CCK (Alexa-488 CCK, yellow signal) is located on the membrane of mouse living acinar cells, suggesting binding to the CCK-receptor (left image). This binding was decreased after co-incubation with TLCS, a hydrophobic BA (middle image), but was unchanged when incubated with TUDCA, a hydrophilic BA (right image). (**B**) Total quantification of the fluorescent signals after incubating acini with Alexa-488 CCK showed a decrease in the fluorescence intensity after addition of a hydrophobic BA, but no reduction with a hydrophilic BA. (**C**) The time course data of the intracellular calcium mobilization measured at 30, 45, 60 and 75 s, which peaked at 30 s and was followed by a quick decrease in the calcium changes. CCK quickly induced intracellular calcium mobilization in mouse acini, which was clearly reduced by simultaneously adding TLCS to CCK, but was not significantly different when compared to co-incubating CCK and TUDCA. (**D**,**E**) When we inhibited CCK1R using the specific inhibitor devazepide and induced AP using caerulein, serum amylase and lipase increases were abrogated and the addition of TLCS showed no further inhibitory effect. (**F**) Mouse pancreatic trypsin activity was also quenched by devazepide. (**G**,**H**) In parallel, both macroscopic observation and histopathologic images demonstrated edema and injury of the pancreas, which were almost absent after devazepide treatment. No additional reduction was observed with TLCS when mice received devazepide beforehand. All results were based on at least 5 animals per group. Statistically significant differences for more than 3 groups were tested via one-way ANOVA followed by Tukey’s multiple comparison test, and significance levels of *p* < 0.05 are marked by an asterisk. ns: non significant.

Our findings underline the differential effects of hydrophobic and hydrophilic BAs in AP and their dependence on the stimulus and the presence of CCK. Since this peptide hormone binds to the G-protein-coupled CCK1 receptor on pancreatic acinar cells, causing multiple cellular signaling functions [26], we next wanted to clarify to what extent BAs act on CCK1R in acinar cells. When we incubated freshly isolated mouse acini with Alexa-488-labeled CCK, we could visualize the binding of the CCK to its receptor via immunofluorescence. The co-incubation of acini with 500 µM TLCS drastically reduced the fluorogenic signal from acini, suggesting that TLCS had affected the binding of CCK1R on the cellular surface with Alexa-488 CCK. When 500 µM TUDCA was added instead of TLCS, the signal intensity on the surface was similar to the situation wherein only Alexa-488 CCK stimulation was used (Figure 7A). Quantification of the total fluorescence intensities derived from fluoroscopy confirmed our observations (Figure 7B) and suggests decreased binding between CCK1R on the membrane of living acini and CCK in the presence of hydrophobic BAs. Moreover, co-incubation of isolated acini with TLCS and CCK reduced intracellular calcium mobilization compared to CCK alone (Figure 7C). Distinguishably, the hydrophilic bile acid TUDCA affected neither CCK1R-CCK binding nor intracellular calcium mobilization. When we pre-treated C57BL/6J mice with devazepide, a CCK1R inhibitor, at a dose of 1 mg/kg bodyweight, and induced AP using caerulein, serum amylase and lipase activities were abrogated and the addition of TLCS did not further decrease the activity (Figure 7D,E). Similarly, trypsin activity was quenched by devazepide (Figure 7F). In parallel, both macroscopic inspection and histopathologic evaluation in caerulein-treated mice showed edema and injury of the pancreas, which was almost absent after devazepide application and could not be further attenuated by TLCS (Figure 7G,H). These results support the hypothesis that TLCS acts via an interaction with the CCK1 receptor, leading to impairment of the intracellular signaling pathways for AP.

## 3. Discussion

Being the leading cause of AP, the biliary origin has been extensively investigated in several studies, but so far, they have almost exclusively been based on the retrograde infusion of BAs into the pancreatic duct [17,27]. To the best of our knowledge, systematic analyses of systemically administered BAs focusing on their impact in AP and investigation of their underlying mechanisms are still lacking. Secondly, a large number of cholestatic disorders are caused not only by obstruction of the bile ducts but also by hepatocellular damage, and lead to an increase in serum BA concentrations [28]; however, their potential impact on AP has not been elucidated so far, which might even enhance the necessity to focus on their systemic role, as well. In the present study, we were able to demonstrate that systemic BAs modulate the severity of AP depending on its pathogenesis and the hydrophobicity of BAs. While hydrophobic BAs aggravate severity under disease conditions that are independent of CCK or its analogues, they reverse CCK-induced injury based on an interaction with the CCK receptor on acinar cells.

In isolated living acini, we confirmed previous studies [16,18], which showed that TLCS and LCA can induce significant activation of pancreatic enzymes. Following pre-incubation with these strongly hydrophobic BAs, concomitant CCK application reverted intracellular zymogen and lysosomal protease activation, while this was not the case with hydrophilic BAs. These BA-dependent differences seem to be related to their ability to interact with CCK receptors, namely CCK1R, which is abundantly expressed on acinar cells and has the highest affinity to the octapeptide CCK [29,30]. Receptor binding is markedly reduced in the presence of hydrophobic compared to hydrophilic BAs, as we demonstrated using CCK labeled with the fluorophore Alexa-488 that was detectable by fluorescence microscopy. As the activation of G-protein-coupled receptors led to a release of intracellular Ca^2+^, a crucial regulator of pancreatic acinar cell secretion [31,32], we further assessed intracellular calcium release and confirmed a reduction in Ca^2+^ mobilization, which was less pronounced after incubation with TLCS but remained steady after TUDCA. It is noteworthy that the TLCS-induced decrease in intracellular Ca^2+^ released by CCK was not strong, so additional intracellular mechanisms may also lead to a decrease in protease activation and pancreatic damage. Our BA concentrations were in the same range as others have used in previous studies [16,18] and isolated acini did not show signs of cellular necrosis upon TLCS stimulation. Therefore, we assume that the TLCS-related reduction in intracellular protease activation in CCK-stimulated acini is primarily based on alterations of intracellular signaling and not a consequence of cytotoxicity.

The impact of BAs on CCK1R function was studied by Desai et al. in Chinese hamster ovary (CHO) cell lines expressing CCK1R. They showed an inhibitory effect with hydrophobic taurochenodeoxycholic (TCDC) acid but not with hydrophilic tauroursodeoxycholic acid (TUDCA), and proposed a direct interaction of BAs with the CCK1 receptor, likely at the same site where cholesterol binds, leading to a conformational change in the helical bundle domain [33]. The intracellular calcium responses following CCK stimulation and BA exposure were also delayed by TCDC, but not TUDCA, similar to our results.

The translation of ex vivo findings into in vivo models that, at least partly, are based on CCK or its analogues confirmed our observations of the attenuating effects of hydrophobic BAs. In both caerulein-induced and BPDL pancreatitis, which caused an elevation of CCK, the severity of pancreatitis was decreased by hydrophobic BAs but not by the hydrophilic TUDCA. Previous studies reported that a lack of BAs in the duodenum, as seen after obstruction of the distal bile duct, triggers a feedback regulation to produce more endogenous CCK [34]. Our results are in line with this observation as CCK strongly increased in mice that underwent combined pancreatic and bile duct ligation in comparison to sham-operated or only pancreatic duct-ligated mice, where significant CCK-elevation was absent. These findings further confirm our observations from ex vivo models and propose a modulating effect of BAs on AP severity in vivo, depending on their hydrophobicity and the presence of CCK in the initial phase of the disease.

Depending on the location where BAs reach acinar cells, a variety of both receptor and transporter-mediated cellular signaling mechanisms contribute to pancreatic damage. In rat pancreatic acinar cells, Kim et al. showed evidence for the expression of the BA transporter molecules Na+/taurocholate co-transporting polypeptide (NTCP) and organic anion transporting polypeptide 1 (OATP1) on the luminal and basolateral site, respectively; these are both capable of mediating BA influx into acinar cells [35]. BAs may therefore enter acinar cells not only via reflux to the apical pole but also because of interstitial leakage or systemic application at the basolateral site. This route of internalization might explain why TLCS increases pancreatic injury in L-arginine or pancreatic duct ligation-induced pancreatitis. L-arginine is the major amino acid of histones, and Guo et al. found that extracellular histones could inhibit the impact of CCK 20 pM on rat pancreatic acini, thus contributing to the secretory blockade [36,37]. It would be interesting to study, in the future, the effect of adding both L-arginine and CCK simultaneously, and possibly find the links between BAs, L-arginine and extracellular histones in AP. Interestingly we additionally observed a protective function of hydrophilic TUDCA. The underlying reason is still unclear but might include: a reduction in endoplasmic reticulum stress, mitochondrial damage in acinar cells and alterations in the gut microbiome, as reported in previous studies [21,38]. The gut microbiome has an emerging role in the metabolism of BAs [39] and in pancreatitis [40]. Studies about the interactions between BAs, the gut microbiome and acute pancreatitis would be a promising approach. Further investigations will also have to clarify whether treatment with hydrophilic BAs may eventually be helpful in preventing or attenuating AP in humans when the etiology and mechanisms are unrelated to CCK.

There are limitations to our work. Firstly, the low number of mice, consisting of four animals in some control groups, that were either untreated, underwent sham operations or received TLCS and TUDCA alone, could limit the validity of the results. In the experimental design, we calculated the number of animals based on a ‘resource equation’ approach [41,42], with a relatively low number of mice in some groups. Secondly, different application routes for bile acids were used, as TLCS was injected intravenously while TUDCA and LCA were administered intraperitoneally. Nevertheless, the TBA concentrations in pancreas homogenates increased similarly. Moreover, previous work [43] reported comparable effects on hepatic CYP-linked mono-oxygenase activities following i.v. or i.p. injection. However, further research will be necessary to clarify the importance of the application route of drugs or compounds in AP. Thirdly, this study mainly concentrated on only two hydrophobic BAs and one hydrophilic BA in AP, which might not be fully representative of all hydrophobic and hydrophilic BAs, respectively. Additional studies using a broader BA spectrum and different models of AP will be necessary for a comprehensive understanding of the role of BAs in AP.

In conclusion, our results indicate that systemic BAs can modulate the severity of AP, which seems to be dependent on the biochemical properties of BAs such as their hydrophobicity and the pathogenesis of AP. The influence of hydrophobic BAs on the CCK1 receptor’s binding emerged as a central mechanism.

## 4. Materials and Methods

### 4.1. Chemicals and Materials

Amylase (11876473316) and lipase (11821792216) kits were purchased from Roche Diagnostics GmbH (Rotkreuz, Switzerland). Cathepsin B substrate (AMC-Arg2, I-1135.0250) was from Bachem (Bubendorf, Switzerland). Non-sulfated CCK 26-33 amide fluorescence-labeled (Alexa-488) was ordered from Thermo Fisher Scientific (Carlsbad, CA, USA). Cell Meter™ No Wash and Probenecid-Free Endpoint Calcium Assay Kit (36312) was ordered from AAT Bioquest (Sunnyvale, CA, USA). Cholecystokinin quantification EIA Kit (RAB0039), Caerulein (17650-98-5) and L-arginine (A5006) were sold by Sigma-Aldrich Chemie GmbH (Merk). Trypsin substrate (R-110 BZIPAR, 10208) was ordered from Bio Trend (Pambio-Noranco, Switzerland). Chymotrypsin substrate (Suc-Ala-Ala-Pro-Phe-AMC, I-1465.0250) was bought from Bachem (Bubendorf, Switzerland). Collagenase (14007) from Clostridium histolyticum (EC.3.4.24.3) was provided by Serva (Heidelberg, Germany). LDH Cytotoxicity Assay Kit (601170) and taurolithocholic acid-3 sulfate (18468) were obtained from Cayman Chemical (Ann Arbor, MI, USA). Tauroursodeoxycholic acid (S3654) was obtained from Selleck Chemicals (Houston, TX, USA). Total BA fluorogenic kit (MET-5005) was a product from Cell Biolabs (San Diego, CA, USA).

### 4.2. Isolation and Stimulation of Mouse Pancreatic Acini

Acini from wildtype C57BL/6J mice were freshly prepared from the pancreas based on collagenase digestion, as described previously [6]. A cell medium containing Dulbecco’s Modified Eagle’s Medium (DMEM), BSA 2% and 10 mM 4-(2-hydroxy-ethyl)-1-piperazine ethanesulfonic acid (HEPES) was freshly prepared. A fresh pancreas was dissected carefully using scissors and forceps. Blood, fat and connective tissue were removed and the clean pancreas was then immediately and gently sheared into small pieces under 1 mm in size in a glass flask containing cell medium and collagenase (1 mg/mouse pancreas). The above process was repeated 2 times with freshly changed medium, and during the 15 min interval, the cells were incubated in a water bath with a shaking speed of 90 rpm for dissociation of the cells. The sheared pancreatic pieces were gently suspended 5 times through 1 mL pipette tips with gradually decreasing diameters followed by filtering twice using single-use, double-layered muslin gauze. The filtered cells were centrifuged for 90 s at 293× *g*, the supernatant was removed and fresh medium was added immediately. Cells were resuspended using a 10 mL pipette and rested for 30 min at the same temperature and buffer conditions in a shaking water bath at 45 rpm. During the whole process, cells were maintained in the oxygenated medium at 37 °C. After isolation, we obtained acini with viability of more than 90%, which were ready for further assays. For the in vitro experiments, BAs were dissolved in DMSO and added to freshly isolated acini at a final concentration of 500 µM. Acini were stimulated with 1 µM CCK for 30 min. Intracellular enzyme activation was determined in a cell medium system at pH 7.4 containing 24.5 mM HEPES, 96 mM NaCl, 11.5 mM glucose, 6 mM KCl, 1 mM MgCl_2_, 0.5 mM CaCl_2_, 2.5 mM NaH_2_PO_4_, 5 mM sodium fumarate, 5 mM sodium glutamate, 5 mM sodium pyruvate and 1% BSA and DMEM.

### 4.3. CCK1 Receptor-Binding Assay

After the resting phase, freshly isolated acini were incubated with 1 µM CCK 26-33 amide fluorescence-labeled (Alexa-488), obtained from Thermo Fisher Scientific (Carlsbad, CA, USA), with or without different BAs at a concentration of 500 µM in a water bath, at 37 °C, with gentle shaking 45 rpm for 30 min. Incubated cell medium was then centrifuged at 1000 rpm for one minute and the supernatant was completely removed and very gently washed with PBS to rule out unbound fluorescence. The pellet was re-suspended in measuring buffer and the remaining fluorescence was quantified at an excitation wavelength of 490 nm, and an emission wavelength of 525 nm. Additionally, living cells were observed under fluorescence microscopy for visualization of the binding between CCK-488 and its receptor on the membranes of acinar cells.

### 4.4. Protease Activation Assays

Trypsin and cathepsin B in isolated acini were measured kinetically in a measuring buffer containing 11.5 mM glucose, 96 mM NaCl, 1 mM MgCl_2_, 6 mM KCl, 2.5 mM NaH_2_PO_4_, 0.5 mM CaCl_2_, 5 mM Na fumarate, 5 mM Na glutamate, 5 mM Na pyruvate, 24.5 mM HEPES and 1% BSA at pH 7.4. The substrates for cathepsin B and trypsin were 10 µM AMC-Arg2 and 10 µM R110-IPA, respectively. In acinar cells and whole pancreatic homogenates, chymotrypsin activity was measured using the substrate Suc-Ala-Ala-Pro-Phe-AMC, and trypsin activity was measured using R-110 BZIPAR. Activities of the enzymes were measured for one hour at 37 °C, kinetically, in a buffer (pH 8.0) containing 5 mM CaCl2 and 100 mM Tris. Fluorometric measurements were carried out in a FLUOStar Omega fluorometer. For AMC-based substrates, the setting was a 380 nm excitation wavelength and 460 nm emission wavelength, and for R110-based substrates, the wavelengths of excitation and emission were 485 nm and 520 nm, respectively. Enzymatic activities were normalized to protein content, which was measured via Bradford assay. All measurements were performed in triplicate.

### 4.5. Quantification of Intracellular Calcium Mobilization in Living Acinar Cells

Total intracellular calcium mobilization was quantified by measuring the fluorescence intensity within one minute after preparing the acini, according to the protocol of the Cell Meter™ No Wash and Probenecid-Free Endpoint Calcium Assay Kit provided by AAT Bioquest (Sunnyvale, CA, USA). Briefly, we firstly added 100 µL/well of Fluo-8E™ AM dye-loading solution into the 96-well plate, with 100µL mouse acini already prepared in each well. Then, we incubated the dye-loading plate in a 5% CO_2_ incubator at 37 °C for 45 min. Meanwhile, we prepared the calcium stimulator solution (CCK and BAs). Finally, we added 50 µL of the prepared stimulator and ran the calcium flux assay immediately, measuring the fluorescence intensity using a FLUOStar Omega fluorometer (BMG Labtech GmbH, Ortenberg, Germany) with the bottom read mode at an excitation/emission of 490/525 nm.

### 4.6. Amylase and Lipase Measurement

Serum amylase and lipase activities were measured kinetically over 30 min via photometric assays using kits from Roche Diagnostics GmbH (Rotkreuz, Switzerland), with absorbance at 405 nm and 570 nm wavelengths, respectively. 

### 4.7. Measurement of Propidium Iodide Exclusion and Release of LDH

Propidium iodide exclusion was used to determine necrosis, and cytotoxicity was quantified by LDH release from acini using the Cytotoxicity Assay Kit (601170), according to the instructions of the manufacturer.

### 4.8. Myeloperoxidase Measurement

Lung tissue was homogenized on ice in a buffer containing 20 mM KH_2_PO_4_ at pH 7.4 and centrifuged at 10,000× *g* for 10 min at 4 °C. The pellet was resuspended in 50 mM KH_2_PO_4_ extraction buffer (pH 6.0) containing EDTA, PMSF and hexadecyltrimethylammonium bromide (5%); frozen in liquid nitrogen and thawed in 4 cycles with gradually smaller pipette tips; sonicated twice; and centrifuged at 10,000× *g* for 10 min at 4 °C. Myeloperoxidase (MPO) activity was measured in 50 mM KH_2_PO_4_ extraction buffer (pH 6.0) containing 0.15 mM H_2_O_2_ and 0.53 mM o-dianisidine using a SpectraMax Spectrophotometer (Molecular Devices, Sunnyvale, CA, USA) at 460 nm and at 30 °C over 10 min. The results were calculated after dividing by the protein amount of the corresponding samples.

### 4.9. Measurement of Total Bile Acid

The total BA in the serum was quantified using a Total Bile Acid Assay Kit (MET-5005, Cell Biolabs, San Diego, CA, USA) using a FLUOStar Omega fluorometer (BMG Labtech GmbH, Ortenberg, Germany) following the protocol of the manufacturer at an excitation/emission wavelength of 560/590 nm. Briefly, pancreas tissues were homogenized via ultra-sonification 2 times at 100% power for 10 s each in cold PBS, and centrifuged at 10,000× *g* at 4 °C for 10 min. The concentration of TBA was determined in the supernatant using the fluorometer and normalized to the corresponding protein amount. Mouse serum was harvested after centrifugation of whole blood and diluted at least 4 times before measuring.

### 4.10. Histopathological Examinations

Pancreases were fixed in 4.5% formaldehyde immediately after harvesting. Paraffin-embedded blocks were used for staining with hematoxylin and eosin. The slides were scanned using the Sysmex Pannoramic MIDI II slide scanner (Sysmex Europe SE, Norderstedt, Germany) for imaging. Damage was assessed using a modified score adapted from Niederau et al. [44]. The extent of edema, including the cell-free areas, was quantified by the percentage of the total areas using QuantCenter software version 2.2.1.88915 from Sysmex.

### 4.11. Animal Models

Wildtype C57BL/6J mice, 8–10 weeks old and weighing 23–27 g, were purchased from Janvier Labs (Le Genest-Saint-Isle, France) and were kept under standard conditions of temperature and humidity in ventilated cages under 12 h day/night cycles, with food and water provided ad libitum. All mice were fasted equally 8 h prior to the experiments. The study design and all protocols for animal care and handling were approved by the Institutional Animal Care and Use Committee of the University of Greifswald (Reg. No.: 7221.3-1-001/20). Mice were treated with BA dose of 50 mg/kg body weight in assigned groups half to one hour before the induction of AP. We used different BAs, including hydrophobic and hydrophilic compounds, to see the different effects that may result from their biochemical features. Due to their solubility and the limited volume that we can inject into the tail vein of a mouse, hydrophobic BA TLCS (10 mg/mL) was injected i.v. while hydrophilic TUDCA (5 mg/mL) and hydrophobic LCA (1 mg/mL) were injected i.p. after dissolving in PBS at 37 °C. Due to the very low solubility of LCA and the similar effects with TLCS, we further focused on TLCS in our experiments after the caerulein model. 

**Figure 8:**
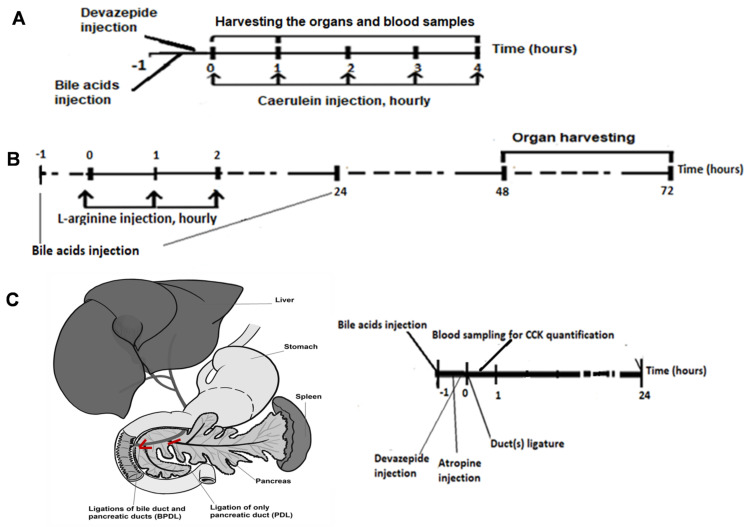
**Animal models for AP to test the effect of systemic BAs.** (**A**) Caerulein model: BAs were injected 30–60 min prior, and devazepide, if utilized, was injected 15 min before the first dose of caerulein. Caerulein was administrated hourly and intraperitoneally in a dose of 50 µg/kg body weight. The organs were harvested at 4 h (half-life time of devazepide). (**B**) L-arginine model: BAs were added one hour before and 24 h after the first injection of L-arginine, which was injected intraperitoneally (total dose 10 g/kg body weight), divided to three injections hourly. The mice were humanely killed via cervical dislocation at 72 h. (**C**) Duct ligation model: BAs were administrated half to one hour prior to ligature. After laparotomy, ligature of both distal common bile duct and main pancreatic duct (BPDL) or ligation of only one distal part of the main pancreatic duct (PDL) was carried out. The blood was withdrawn 30 min after duct ligation to quantify the endogenous CCK concentration. Organs were harvested 24 h after the surgical duct ligation. BA dose: 50 mg/kg body weight.

Caerulein-induced pancreatitis was induced by intraperitoneal injections of the cholecystokinin analogue caerulein (50 µg/kg/body weight) at hourly intervals for up to 4 h. Mice were humanely killed via cervical dislocation to harvest the samples 1 h and 4 h after the first injection of caerulein (Figure 8A).

In L-arginine pancreatitis, mice received L-arginine i.p. at a total dose of 10 g/kg of body weight, divided into 3 injections at hourly intervals. Control mice received PBS in parallel. Organs were harvested after 72 h (Figure 8B).

Duct ligation models were based either on the ligation of solely the main pancreatic duct [45] or the combined ligation of the bile and major pancreatic ducts, as described previously [46]. BAs were prepared as in the caerulein and L-arginine models. After laparotomy, either the confluence of the distal common bile duct and the main pancreatic duct was closed, called the bile-and-pancreatic duct ligation (BPDL) model, or only the distal part of the major pancreatic duct was ligated, called the pancreatic duct ligation (PDL) model (Figure 8C). For CCK quantification, blood samples were collected 30 min after surgery. The mice were humanely killed via cervical dislocation 24 h after ligature.

### 4.12. Statistics

Statistical analysis was performed using GraphPad Prism version 8.4.3 (GraphPad Software, San Diego, CA, USA). Data were presented as mean ± standard deviation (SD) for each group of animals. We used one-way ANOVA followed by Tukey’s multiple comparison test for comparisons of more than 3 groups. Differences were considered significant when *p* < 0.05.
